# Utilization of Personalized Machine-Learning to Screen for Dysglycemia from Ambulatory ECG, toward Noninvasive Blood Glucose Monitoring

**DOI:** 10.3390/bios13010023

**Published:** 2022-12-25

**Authors:** I-Min Chiu, Chi-Yung Cheng, Po-Kai Chang, Chao-Jui Li, Fu-Jen Cheng, Chun-Hung Richard Lin

**Affiliations:** 1 Department of Computer Science and Engineering, National Sun Yat-Sen University, Kaohsiung 804, Taiwan; 2 Department of Emergency Medicine, Kaohsiung Chang Gung Memorial Hospital, Kaohsiung 833, Taiwan

**Keywords:** machine learning, dysglycemia, blood glucose, ECG, personalized medicine, noninvasive blood glucose monitor

## Abstract

Blood glucose (BG) monitoring is important for critically ill patients, as poor sugar control has been associated with increased mortality in hospitalized patients. However, constant BG monitoring can be resource-intensive and pose a healthcare burden in clinical practice. In this study, we aimed to develop a personalized machine-learning model to predict dysglycemia from electrocardiogram (ECG) data. We used the Medical Information Mart for Intensive Care III database as our source of data and obtained more than 20 ECG records from each included patient during a single hospital admission. We focused on lead II recordings, along with corresponding blood sugar data. We processed the data and used ECG features from each heartbeat as inputs to develop a one-class support vector machine algorithm to predict dysglycemia. The model was able to predict dysglycemia using a single heartbeat with an AUC of 0.92 ± 0.09, a sensitivity of 0.92 ± 0.10, and specificity of 0.84 ± 0.04. After applying 10 s majority voting, the AUC of the model’s dysglycemia prediction increased to 0.97 ± 0.06. This study showed that a personalized machine-learning algorithm can accurately detect dysglycemia from a single-lead ECG.

## 1. Introduction

Blood glucose (BG) monitoring and control are extremely important in global health care. The incidence of hyperglycemia in critically ill patients is high. Researchers have reported that poor sugar control is associated with increased mortality in admitted patients [1,2,3,4]. In critically ill patients, continuous glucose monitoring can prevent acute complications such as severe hypoglycemia [5,6]. BG is typically measured using a glucose meter after a lancing device and a test strip have been used to obtain a blood sample. Repeated blood glucose monitoring can be resource-consuming and pose a healthcare burden in clinical practice. Drawing blood is also a painful and distressing experience, leading to low adherence to general practice in home healthcare [7,8].

Continuous glucose-monitoring devices have been developed for BG measurements [9]. In place of the finger-prick test, it uses a glucose probe inserted into the subcutaneous tissue to achieve the automatic monitoring of glucose in the interstitial fluid per 5–10 min. However, the lifetime of the probe is usually limited to 3–14 days. Therefore, the development of an affordable, noninvasive approach is necessary. Several noninvasive continuous glucose monitoring techniques have been developed, including methods that utilize Raman spectroscopy, fluorescence technology, mid-infrared spectroscopy, near-infrared spectroscopy, optical coherence tomography, optical polarimetry, and microwave planar resonant sensor [10,11,12]. Although such devices have yielded promising results, their equipment should be improved to make them more accurate, convenient, comfortable to wear, and available for personalized use at home.

The idea of using electrocardiogram (ECG) features to determine BG levels has been previously proposed. Previous research has suggested that hyperglycemia and hypoglycemia are both correlated with a prolonged QT interval and decreased heart rate variability on ECG [13,14,15]. However, it is still difficult to identify dysglycemia based on ECG because numerous differential diagnoses should be considered when interpreting ECG findings.

Owing to an increase in storage ability and computing power, machine learning has recently begun to evolve in the medical field. Machine-learning-assisted ECG interpretation has shown promising results in distinguishing cardiac arrhythmia and predicting certain metabolic illnesses such as hyperkalemia [16,17,18]. In the past few years, there has also been a trend to use machine learning to predict hypoglycemic episodes from ECG, which has demonstrated its potential [19]. However, a recent meta-analysis showed that machine learning had a sensitivity of only 0.72–0.86 in predicting hypoglycemia in patients with diabetes mellitus, which is still insufficient for clinical applications [20].

In this study, we aimed to develop a personalized machine-learning model to predict dysglycemia, including hyperglycemia and hypoglycemia, based on ECG data. We believe that this model can improve the clinical practice of blood glucose monitoring, optimize the use of human resources, and improve the quality of life of patients.

## 2. Materials and Methods

The data collection and protocols used in this study were approved by the Institutional Review Board of the Chang Gung Medical Foundation (202100362B0). The data supporting this study’s findings are available in the Medical Information Mart for Intensive Care (MIMIC)-III Waveform Database Matched Subset (http://doi.org/10.13026/c2294b, accessed on 17 September 2022) [21].

### 2.1. Dataset Collection and Inclusion Criteria

In this study, data from the Waveform Database Matched Subset of the Medical Information Mart from Intensive Care III (MIMIC-III) were used. The dataset contained the recordings of 22,317 waveforms and 22,247 numerics for 10,282 distinct ICU patients admitted to the critical care units of medical centers in the United States between 2001 and 2012 [22]. These recordings typically include digitized signals such as ECG, arterial blood pressure, and respiration data, as well as periodic measurements such as heart rate, oxygen saturation, and blood pressure values. The ECG signals contained in the dataset are typically lead I, lead II, or lead V signals. This subset represents the records for identifiable patients whose clinical records are available in the matched clinical database.

In this study, we considered patients in the MIMIC-III database for whom at least 20 BG records were made during a single hospital admission. Patients with atrial fibrillation or an implanted pacemaker were excluded from the study. Patients with less than five dysglycemia data points during a single hospital admission were also excluded. We defined two classes of BG levels: dysglycemia for BG > 200 mg/dL or BG < 70 mg/dL, and euglycemia for BG between 80 and 180 mg/dL. ECG signals corresponding to BG values in the ranges of 70–80 and 180–200 were not considered during training in this study to ensure that no consecutive heartbeats would be considered as both hypoglycemia and euglycemia or both euglycemia and hyperglycemia.

### 2.2. Training and Validation Dataset

We randomly selected 10 euglycemic BG data from included patients, along with the corresponding lead II ECG records, as the training dataset for developing a one-class machine-learning model. We then randomly selected five euglycemic and five dysglycemic BG data points from the rest, along with their corresponding ECG records, as the validation dataset. We defined a corresponding ECG of one BG data point as the signal within a 10 min period before the storage time of the BG record. So, the training dataset for each patient would be 10 ECG strip of total 100 min.

### 2.3. ECG Segmentation and Feature Extraction

The MIMIC-III database contains one-dimensional digital ECG records with a resolution of 125 samples per second and an amplitude quantization of microvolts (*μV*). These signals were used in our study to develop a personalized model for non-invasive BG monitoring. We retrieved the corresponding ECG signals and segmented each ECG record into multiple heartbeats with 1 s segments based on the R-peak position in the ratio of 2:3. Therefore, each heartbeat segment contained 50 samples and 75 samples before and after the R-peak position, respectively. R-peak was capture using BioSPPy 0.6.1 software, which receive input of 10 min ECG strip and output the location of R-peak position from multiple cardiac cycles [23]. The segmented heartbeat was first manually inspected to exclude those with a high level of ECG signal noise. This process helped to reduce model overfitting and deviation as a result of noisy data.

**Figure 1:**
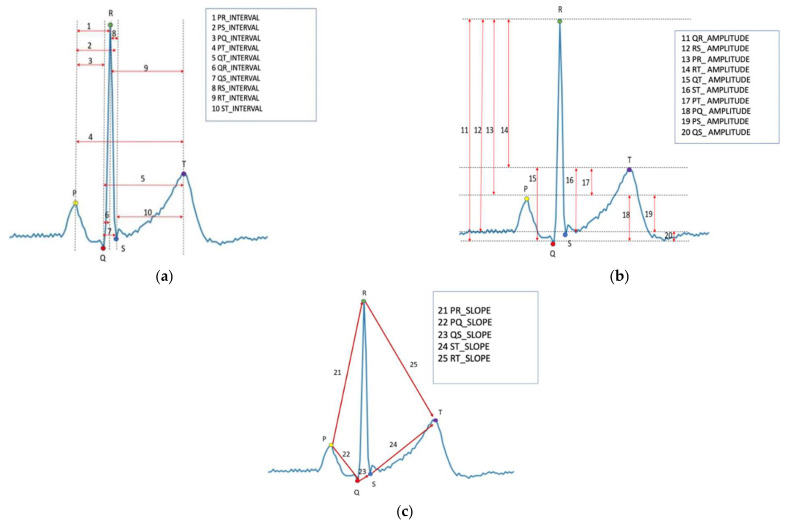
The extracted features from one heartbeat ECG cycle. (**a**) Features of 10 different intervals, (**b**) features of 10 different amplitude, (**c**) features of 5 different slope gradients.

Following heartbeat segmentation, we managed segmented ECG by PyWavelets software to generate a signal amplitude change list for each cardiac cycle [24]. We then identified the position of P, Q, S, and T point base on amplitude difference base on their order of location to R-peak. Afterward, ECG features related to the P-Q-R-S-T point position correlations, which included the amplitude, interval, and slope gradient between two of the five points in one heartbeat ECG cycle, were extracted. Furthermore, ten interval features (Figure 1a) and 15 amplitude features (Figure 1b,c) were extracted. The R-peak interval to the next heartbeat was collected as an input feature.

### 2.4. Machine-Learning Algorithm

In this study, we developed a one-class support vector machine (Oc-SVM) algorithm to predict dysglycemia based on ECG features. SVM is a machine-learning algorithm that can create a nonlinear decision boundary by projecting data through a nonlinear function onto a space with a higher dimension. Thus, the data points that could not be separated by a straight line in their original space were shifted to a feature space where there could be a straight hyperplane demarcating the data points of two classes. When the hyperplane was projected back onto the input space, it had the form of a nonlinear curve. The Oc-SVM is typically applied to specific tasks, such as anomaly detection or fault detection, where positive cases are difficult to collect during the training process. In our study, it was trained using ten normal BG datasets with their corresponding ECG heartbeats.

### 2.5. Statistical Analysis

Data are presented as mean (standard deviation (SD)) for continuous variables, proportions for nominal variables, and median (interquartile range) for ordinal variables. The model was evaluated based on accuracy, sensitivity, specificity, and area under the receiver operating characteristics curve (AUC). From the clinical perspective, sensitivity was considered more relevant than specificity because it showed how accurately hypoglycemia and hyperglycemia events were identified. Thus, when comparing different models, the sensitivity was considered more important.

When training the model, the inputs of the SVM model and its output prediction were based on segmented heartbeats. In clinical applications, predicting dysglycemia for every heartbeat is undesirable, and the result may fluctuate, which makes it difficult to follow. Generating a prediction every 10 s, which is represented by a standard ECG, is more feasible. Therefore, we also evaluated the model’s performance in a 10 s window of time by taking the majority class of the heartbeat predictions in that specific timeframe.

## 3. Results

In this study, we used 50 patients from the MIMIC-III database for the analysis (Table S1). Their median age was 64 (55–72) years old, with 27 (54.0%) being male. Majority of them were white (58.0%). Many of them were on ICU admission diagnosed with cardiovascular disease (26.0%), neurological disease (22.0%), respiratory disease (14.0%), infectious diseases (12.0%), gastrointestinal disease (8.0%), metabolic disease (8.0%), and others. Other demographic characteristics are presented in Table 1.

To clarify the relationship of ECG features to BG change, we reported the statistical differences of selected ECG features between normal and dysglycemia from the training data of included patients. In general comparison, ECG manifestation in dysglycemic status was associated with P–R, P–Q, R–S, S–T, and Q–T interval. R–R interval was shortened (0.74 ± 0.52 vs. 0.66 ± 0.50, *p* < 0.001) in dysglycemia indicated higher heart rate. Compare to normal, dysglycemia was also associated with lower amplitude of R wave, including Q–R amplitude (0.68 ± 0.46 vs. 0.56 ± 0.43, *p* < 0.001) and R–S amplitude (0.75 ± 0.56 vs. 0.71 ± 0.49, *p* < 0.001). Other characteristics of ECG morphology to BG change are shown in Table 2.

During training, each patient was assigned a model weight based on the training data. Individual Oc-SVM models with the same hyperparameters were developed for the patients. The kernel function selected was a linear SVM. The “ν” argument indicates an upper bound on the fraction of training errors and a lower bound of the fraction of support vectors, and was set to 0.75 to provide better sensitivity for dysglycemia during the prediction.

**Figure 2:**
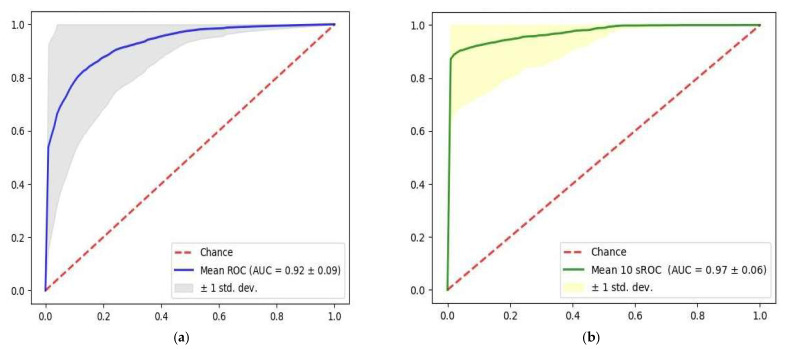
Receiver Operating Curve of model prediction based on (**a**) single heartbeat and (**b**) 10 s majority voting.

The prediction performance of the developed models for all the patients is presented in Table S2. The prediction performance for a single heartbeat and for a 10 s strip are presented in Tables S1 and S2. The model prediction for dysglycemia from a single heartbeat had an AUC level of 0.92 ± 0.09 (Figure 2a), with a sensitivity of 0.92 ± 0.10 and specificity of 0.84 ± 0.04. The positive predictive value (PPV) for a single heartbeat was 0.85 ± 0.03, and the negative predictive value (NPV) was 0.92 ± 0.09. Based on 10 s majority voting, the AUC of the model prediction for dysglycemia increased to 0.97 ± 0.06 (Figure 2b). Other performance measurements are presented in Table 3.

Table 4 demonstrates the F-score as feature importance of the developed Oc-SVM model of all the included patients. In linear SVM, the model is trained to find a hyperplane boundary that separates the classes as best as possible. The coefficients are the weights represent this hyperplane, by giving you the coordinates of a vector which is orthogonal to the hyperplane. The longer the vector, the bigger the importance of feature. The most important ECG feature for predicting dysglycemia was the R–R interval, followed by the R–S, P–T, Q–R, Q–R, S–T, R–T, and R–S intervals.

## 4. Discussion

In this study, we aimed to develop a personalized machine-learning algorithm to recognize dysglycemia from an ECG recording using only the lead II ECG record. Using personalized data, we found that the Oc-SVM model could accurately predict dysglycemia from a single heartbeat, with a high AUC of 0.92, which increased to 0.97 when using 10 s majority voting. In our exploration of the research, we also assessed the use of a one-class neural network. We found that the network architecture, including the number of layers and the number of neurons in each layer, had to be adjusted for different patients in order to achieve optimal performance. However, this made future deployment more difficult, as manual tuning of hyperparameters would be required for each individual. Additionally, the performance of the one-class neural network was not superior to that of the Oc-SVM method. As a result, we only reported the results of the Oc-SVM in our study.

The most common method for BG testing is finger-stick glucose monitoring, which is not only invasive but also cumbersome and expensive, leading to poor patient compliance for glucose measurement [25]. In addition, it does not allow continuous monitoring. Continuous glucose-monitoring devices have been developed to replace the finger-prick test. So far, the development of a more affordable, noninvasive approach is still in progress. Several noninvasive continuous glucose monitoring techniques have been developed and show promise, but their equipment needs to be improved for accuracy, convenience, comfort, and availability for personal use at home.

An ECG can reflect significant amount of information about the electrical activity of the heart. In the past decade, wearable noninvasive sensors have been developed for tracking cardiac signals. ECG revealed the function of the cardiovascular system and changes in the sympathetic and parasympathetic nervous systems, which are associated with changes in BG levels. ECG signals can be easily obtained through wearable devices, such as smart watches; thus, the ECG-based approach can be used for real-time monitoring of daily life, especially for high-risk individuals [26].

Our work demonstrated ECG morphology change in dysglycemia that correlates with previous studies (Table 2). Considering interval change, dysglycemia was associated with prolongation of QT interval. This finding was documented in several previous research where QT prolongation was found in both hyperglycemic and hypoglycemic status, and was associated with increasing mortality in critical ill patients [27,28]. Other electrophysiologic alterations created by dysglycemia include ST-segment depression and T-wave flattening, and were also demonstrated by our statistical analysis by showing decreased ST amplitude in dysglycemia. Higher heart rate was also found in dysglycemic status. In previous studies, both hypoglycemia and hyperglycemia allowed a faster heart rate [29]. As a group of data, these values of R-R interval may show the variability of heart rate. Thus, the collection of these ECG features can help in building up a machine-learning model for dysglycemia prediction.

Several methods have been proposed for the use of an ECG to detect dysglycaemic events. Based on an ECG, Ling et al. proposed a hybrid neural logic approach that detected hypoglycemic events with an average sensitivity of 79.07% and a specificity of 53.64% [30]. Using a deep belief network for the detection of hypoglycemic episodes in diabetes patients, San et al. achieved sensitivity and specificity values of 80.00% and 50.00%, respectively [31]. Cordeiro et al. evaluated ECG data from 1119 patients and found that a 10-layer-deep neural network was effective in detecting hyperglycemia, with an AUC of 94.53%, 87.57% sensitivity, and 85.04% specificity [32].

However, this research was conducted to detect hypoglycemia or hyperglycemia only. Very few studies have detected ECG changes indicating the hypoglycemic and hyperglycemic states. Although Nguyen et al. revealed that ECG parameters could be used to identify hypoglycemia and hyperglycemia in patients with type 1 diabetes, they did not develop an AI model to detect dysglycemic events [33]. Cordeiro et al. developed a deep learning model that was able to detect hyperglycemia with an AUC of 0.95, which showed promise [32]. However, there were some differences between their study and ours. First, we collected data from an ambulatory ECG monitor, whereas Cordeiro et al. used simulated lead I data from a wearable device. Second, it was not clear whether Cordeiro et al. mixed cardiac cycle data from same ECG readings in their training and validation sets, which could have inflated their results. In contrast, we strictly separated our training and validation data.

Most of the studies described above attempted to detect dysglycemia through noninvasive monitoring using features extracted from ECG signals from generalized data. To date, only a limited number of studies have been conducted to detect dysglycemia using personalized ECG signals. Porumb et al. demonstrated that a convolutional neural network-based model can detect hypoglycemic events from personalized raw ECG signals recorded using noninvasive wearable devices with a sensitivity of 87.5% and specificity of 81.7% [34]. To the best of our knowledge, the proposed personalized model for simultaneously detecting both hyperglycemia and hypoglycemia is novel. With ECG signal detection using a 10 s window, the model performance yielded high AUC, sensitivity, and specificity values of 0.97, 0.97, and 0.96, respectively. In addition, instead of using a multiple-lead ECG, we used only a single-lead ECG for signal acquisition. Because consumer wearable devices with ECG-reading capabilities, such as smart watches, are becoming easily accessible, this technique can be useful to the general public.

BG concentration affects the electrical activity of the heart [35]. Blood heart rate variability (HRV), a representative cardiovascular autonomic function, is considered to be significantly modulated by BG levels. Bekkink et al. demonstrated that hypoglycemic events are related to an increase in the low-frequency (LF)/high-frequency (HF) ratio and a decrease in the square root of the mean standard differences of successive R-R intervals [36]. Amanipour et al. observed a six-fold decrease in the LF/HF ratio with hyperglycemia [37]. The QT interval is also recognized as one of the most common features of cardiopathy assessment in dysglycemia [38]. Robinson et al. demonstrated that hypoglycemia could lead to QTc and QTd lengthening from baselines of ~75 ms and 55 ms, respectively [39]. Among 8277 participants, Arnaud et al. found that severe hypoglycemia was associated with an increased risk of QTc prolongation, independent of other risk factors such as cardiac autonomic neuropathy [40]. Pickham et al. revealed that elevated glucose levels of 140–180 mg/dL corresponded to 2.1 odds of QTc interval prolongation. The odds are 3.8 for glucose levels above 180 mg/dL [28]. In a population-based study, impaired fasting serum glucose levels led to significant QTc lengthening and RR interval shortening, and both phenomena were associated with an increased risk of sudden cardiac death [41]. ECG changes in response to hypoglycemia include an increased QTc interval, decreased PR interval, increased R-wave amplitude, decreased T-wave amplitude, and ST depression [42]. In cases of hyperglycemia, other ECG abnormalities, such as significant increases in the PR interval and shorter mean RR intervals, have been reported [43,44]. For additional information, we ranked the results of the variables by their importance. This would enable a clinical physician to recognize the part of the ECG signal significantly associated with dysglycemia. In our study, the most important ECG feature for predicting dysglycemia was the R-R interval, followed by the R-S, P-T, Q-R, Q-R, S-T, R-T, and R-S intervals (Table 4).

Our study has several limitations. In this study, we developed a personalized Oc-SVM model using a limited number of data points. In real-world applications, it would be necessary to gather multiple samples of dysglycemic ECG signals from a user. Despite being based on an open-source database, our results suggest the potential for non-invasive BG monitoring using this method. Second, although HRV is associated with dysglycemia, it is difficult to measure HRV from a single heartbeat. We used the R-R interval, which reflects heart rate and is inversely associated with HRV, as an input feature, but this may not be sufficient to capture HRV accurately. Third, we did not differentiate between hyperglycemia and hypoglycemia in our model predictions. Previous studies have shown that these conditions may be associated with similar ECG changes, making them difficult to distinguish from each other. In addition, one-class algorithms are suitable for predicting abnormal events from patterns of normal data, but they may not be suitable for differentiating two minority classes, hyper- and hypoglycemia in this scenario. Collecting ECG signals might be challenging owing to their sensitivity to diverse environmental stresses, which could affect the quality of the data. However, wearable devices have been shown to achieve high diagnostic accuracy [45]. Further research should be conducted using the ECG signals from wearable devices.

## 5. Conclusions

We developed a personalized machine-learning algorithm with Oc-SVM that can detect dysglycemia from only lead II ECG records. This noninvasive, continuous monitoring method had a high AUC of 0.97. Since it only requires a single-lead ECG for signal acquisition, it is expected to be easily accessible to the general public. Additionally, ranking ECG features using the Oc-SVM model would allow physicians to identify which components of the ECG signal are significantly associated with dysglycemia.

## Figures and Tables

**Table 1:** Demographics of the included patients.

Variables	Median (IQR)/N (%)
Age, median (IQR)	64 (55–72)
Male, *n* (%)	27 (54.0)
Race	
White	29 (58.0)
Black	10 (20.0)
Asian	2 (4.0)
Latino	2 (4.0)
Height (cm), median (IQR)	172 (163–180)
Weight (Kg), median (IQR)	83.6 (70.2–96.3)
BMI, median (IQR)	27.9 (25.4–29.7)
Diagnosis at admission	
Cardiovascular	13 (26.0)
CNS	11 (22.0)
Respiratory	7 (14.0)
Infectious	6 (12.0)
Gastrointestinal	4 (8.0)
Metabolic	4 (8.0)
Others	5 (10.0)

IQR: Interquartile Range, BMI: Body Mass Index, CNS: Central Nervous System.

**Table 2:** Statistical differences of selected ECG features between normal and dysglycemia.

	Normal	Dysglycemia	*p*-Value
R–R interval (s)	0.74 ± 0.52	0.66 ± 0.50	<0.001
P–Q interval (s)	0.13 ± 0.07	0.16 ± 0.09	<0.001
Q–R interval (s)	0.08 ± 0.06	0.07 ± 0.05	<0.001
R–S interval (s)	0.04 ± 0.03	0.05 ± 0.03	<0.001
S–T interval (s)	0.25 ± 0.08	0.32 ± 0.09	<0.001
P–R interval (s)	0.21 ± 0.09	0.23 ± 0.10	<0.001
Q–T interval (s)	0.37 ± 0.13	0.44 ± 0.15	<0.001
P–Q amplitude (mV)	0.13 ± 0.05	0.15 ± 0.07	<0.001
Q–R amplitude (mV)	0.68 ± 0.46	0.56 ± 0.43	<0.001
R–S amplitude (mV)	0.75 ± 0.56	0.71 ± 0.49	<0.001
Q–S amplitude (mV)	0.07 ± 0.05	0.05 ± 0.04	<0.001
S–T amplitude (mV)	0.64 ± 0.43	0.58 ± 0.34	<0.001
P–R slope (mV/s)	0.61 ± 0.58	0.81 ± 0.79	<0.001
P–Q slope (mV/s)	−1.14 ± 0.53	−1.08 ± 0.58	<0.001
Q–S slope (mV/s)	−0.31 ± 0.27	−0.12 ± 0.08	<0.001
S–T slope (mV/s)	5.92 ± 5.91	4.64 ± 4.95	<0.001
R–T slope (mV/s)	−0.68 ± 0.60	−0.58 ± 0.68	<0.001

s: second; mV: mini-Volt.

**Table 3:** Performance of model prediction based on single heartbeat and 10 s majority voting.

Oc-SVM	AUC	Sensitivity	Specificity	PPV	NPV
Single heartbeat	0.92 ± 0.09	0.92 ± 0.10	0.84 ± 0.04	0.85 ± 0.03	0.92 ± 0.09
10 s	0.97 ± 0.06	0.97 ± 0.09	0.96 ± 0.04	0.96 ± 0.04	0.97 ± 0.09

AUC: Area Under the Receiver operating curve, PPV: Positive Predictive Value, NPV: Negative Predictive Value.

**Table 4:** Feature importance of Oc-SVM model in predicting dysglycemia.

ECG Features	F-Score
R–R interval	591
R–S amplitude	271
P–T amplitude	153
Q–R amplitude	150
Q–T interval	98
S–T slope	97
R–T amplitude	76
R–S interval	76
P–S amplitude	72
P–Q amplitude	69
P–R slope	69
R–T slope	69

The F-score was calculated based on single heartbeat average from included patients.

## Data Availability

The data supporting this study’s findings are openly available in the MIMIC-III Waveform Database Matched Subset (http://doi.org/10.13026/c2294b, accessed on 17 September 2022).

## Supplementary Materials

The following supporting information can be downloaded at: https://www.mdpi.com/article/10.3390/bios13010023/s1, Table S1: Demographics and diagnosis of all included patients; Table S2: The prediction performances of the developed models for all the patients.
