# Supplementary material for: Utilization of Personalized Machine-Learning to Screen for Dysglycemia from Ambulatory ECG, toward Noninvasive Blood Glucose Monitoring
Source: Biosensors (Basel). 2022 Dec 25;13(1):23. doi: 10.3390/bios13010023 (PMC9855414; doi:10.3390/bios13010023)
Supplement: Supplementary file 1 [file biosensors-13-00023-s001.zip › Table S1.pdf]

Appendix 1.

SUBJECT_II	HADM_I	hadm ag	sex	height(cm	weight(kg)	admission_diagnosis
12122	100779	63	M	183	118	TRACHEOBRONCHIOMALACIA
21318	186933	55	F	157	56	TRACHEAL LEAK;RESPIRATORY
23657	125544	32	M	180	87	DEHYDRATION
40013	120406	59	F	157	70.2	CHEST PAIN
40057	160818	54	M	178	75.5	CORONARY ARTERY DISEASE
43737	130527	68	F	163	77	STROKE;TRANSIENT ISCHEMIC
43961	150566	39	F	173	96	PRE OP LIVER
44059	198015	76	F	165	86.6	CHEST PAIN
44789	180262	68	F	161	66.9	SUBARACNOID HEMORRHAGE
44829	118249	73	F	163	74.3	PNEUMONIA
46320	113335	50	F	163	71	T8 SPINAL CORD COMPRESSIOI
48056	196072	66	M		106.5	SUBARACHNOID HEMORRHAG
48388	156310	58	M	188	89.2	ABDOMINAL PAIN
49555	111955	75	F	155	64	VOCAL CORD DISFUNCTION
51078	118253	64	F		72	CHEMO
51385	163265	56	M	155	60.6	AORTIC VALVE VEGETATION
52641	131760	80	M	174	85	HEART FAILURE
54757	112220	41	M	180	86	CHOLANGIOCARCINOMA/SDA
56069	195834	61	M		114	GASTROINTESTINAL BLEED
56264	135431	81	F	160	71	CORONARY ARTERY DISEASE\(
56307	103947	60	F	155	61.8	ALTERED MENTAL STATUS
58526	148559	36	F		92.5	DIABETIC KETOACIDOSIS;ACU'
59085	115300	61	M	183	99.1	CORONARY ARTERY DISEASE
60531	182932	51	M	180	100.4	BRAIN MASS
61619	136328	56	F	170	55.4	CARDIAC ARREST
62232	162039	54	F		64.9	INTRACRANIAL HEMORRHAGE
62795	173748	46	F	168	79	BACK PAIN
63961	160874	67	F	165	75.7	ACUTE RENAL FAILURE;JAUNE
66571	117918	66	M	173	61	SMS OBSTRUCTION
67856	150934	62	F	165	90	? AORTIC DISSECTION
67996	183522	51	M		100	INTRACRANIAL HEMORRHAGE
68391	104896	68	M	183	92.3	CELLULITIS
68453	185604	63	M	175	90.2	FOURNIER'S GANGRENE
72196	112776	75	F		85.7	SMALL BOWEL OBSTRUCTION
73299	190596	54	M	188	100	SEPTIC SHOCK
75138	180499	56	F	165	72	ASTHMA;CHRONIC OBST PULM
76930	139955		M	175	80	S/P CARDIAC ARREST
77220	134568	56	M	178	93.5	NECROTIZING PANCREATITIS
77927	177095		F	165	67	PULMONARY INFILTRATE
80209	152675	79	M		92.3	SEIZURE
80350	113374	67	M		93.7	HYDROCEPHALUS
80536	128948	70	F	157	82.1	ACUTE RENAL FAILURE
81303	172869	64	M	183	74	CORONARY ARTERY DISEASE\(
81593	192553	68	F		118.1	URINARY TRACT INFECTION;P'
81636	145069	68	M	163	50.6	RESPIRATORY FAILURE
82065	137855	40	M	178	97	CARDIOGENIC SHOCK
83962	104127	71	M	183	99.7	JAUNDICE
88696	190494	74	M	164	99	CENTRAL CORD SYNDROME
89303	139801	32	F	147	63.5	C SECTION,REPEAT

93025	163620	M	183	75.9	CONGESTIVE HEART FAILURE;[illegible]

Final diagnose	Final diagnoses_1	Final diagnoses_2	Final diagnoses_3
icd9_code	51919	51851	99731
icd9_code	51883	5070	V4611
icd9_code	25013	5849	5856
icd9_code	41401	51851	43491
icd9_code	41091	78551	51881
icd9_code	43491	V420	5849
icd9_code	57142	4168	5715
icd9_code	41519	5845	78559
icd9_code	430	43491	3314
icd9_code	3849	78552	56723
icd9_code	73008	3241	3441
icd9_code	430	5180	37854
icd9_code	56211	380	41511
icd9_code	389	78552	5130
icd9_code	28419	5845	570
icd9_code	99661	3812	4210
icd9_code	42823	5845	78551
icd9_code	1551	56722	389
icd9_code	5723	5722	45620
icd9_code	41401	570	5845
icd9_code	389	5845	51881
icd9_code	25013	5849	5854
icd9_code	42823	389	78552
icd9_code	1300	3485	3484
icd9_code	42741	41091	570
icd9_code	4329	4019	2724
icd9_code	3369	5845	5724
icd9_code	56789	5724	7044
icd9_code	5570	4474	51851
icd9_code	44101	5184	51881
icd9_code	431	43311	43331
icd9_code	25060	42823	41071
icd9_code	60883	389	99592
icd9_code	5570	5070	48282
icd9_code	389	78552	51881
icd9_code	382	51884	481
icd9_code	7991	4275	3481
icd9_code	5770	452	486
icd9_code	486	51881	42831
icd9_code	34570	5845	5070
icd9_code	3314	34830	53501
icd9_code	5849	389	99592
icd9_code	41401	5856	25041
icd9_code	99931	3812	51881
icd9_code	51881	5849	78550
icd9_code	41071	78551	42821
icd9_code	570	57142	5722
icd9_code	95208	51881	9584
icd9_code	64671	5722	66911

icd9_code	383	42833	5849

Final diagnoses_4	Final diagnoses_5
1120	5849
51919	4786
40391	58881
5781	99731
42741	99931
3485	34292
V462	5768
2764	5854
4359	2760
4820	5849
49121	5119
25002	78060
4821	99591
48282	48242
5734	5722
99591	5856
2764	2761
78552	5109
78559	2869
78551	51852
25022	5070
3371	25063
99592	486
51881	34292
78001	3481
25001	
5185	51189
53551	5762
3842	48282
44323	5849
4375	4019
486	51881
78552	78559
5185	78959
2866	3485
5849	2762
42822	78001
2910	5119
2760	80502
34830	99731
5070	41519
5722	452
25081	40391
78552	5845
48242	34982
43411	51881
5845	2866
5180	5119
5724	3485

51852	2760
