# Supplementary material for: Utilization of Personalized Machine-Learning to Screen for Dysglycemia from Ambulatory ECG, toward Noninvasive Blood Glucose Monitoring
Source: Biosensors (Basel). 2022 Dec 25;13(1):23. doi: 10.3390/bios13010023 (PMC9855414; doi:10.3390/bios13010023)
Supplement: Supplementary file 1 [file biosensors-13-00023-s001.zip › Table S2.pdf]

Appendix 2A.

SUBJECT_ID	Sensitivity	Specificity	PPV	NPV
12122	0.82	0.85	0.85	0.83
21328	0.97	0.84	0.86	0.97
23657	0.92	0.87	0.88	0.92
40013	0.88	0.85	0.85	0.88
40057	1	0.85	0.87	1.00
43737	0.92	0.85	0.86	0.91
43961	0.99	0.86	0.88	0.99
44059	0.87	0.84	0.84	0.87
44789	1	0.83	0.85	1.00
44829	0.82	0.85	0.85	0.83
46320	0.97	0.89	0.90	0.97
48056	1	0.84	0.86	1.00
48388	0.91	0.85	0.86	0.90
49555	0.97	0.85	0.87	0.97
51078	0.96	0.86	0.87	0.96
51385	0.83	0.8	0.81	0.82
52641	1	0.65	0.74	1.00
54757	0.8	0.83	0.82	0.81
56069	0.98	0.85	0.87	0.98
56264	1	0.85	0.87	1.00
56307	0.994	0.84	0.86	0.99
58526	0.89	0.83	0.84	0.88
59085	0.94	0.81	0.83	0.93
60531	0.97	0.84	0.86	0.97
61619	0.99	0.85	0.87	0.99
62232	1	0.91	0.92	1.00
62795	0.8	0.86	0.85	0.81
63961	1	0.84	0.86	1.00
66571	0.9	0.83	0.84	0.89
67856	0.98	0.83	0.85	0.98
67996	1	0.85	0.87	1.00
68391	1	0.85	0.87	1.00

68453	0.76	0.84	0.83	0.78
72196	0.9	0.82	0.83	0.89
73299	0.7	0.86	0.83	0.74
75138	0.99	0.85	0.87	0.99
76930	1	0.85	0.87	1.00
77220	0.7	0.85	0.82	0.74
77927	0.64	0.86	0.82	0.70
80209	0.87	0.86	0.86	0.87
80350	0.94	0.73	0.78	0.92
80536	1	0.85	0.87	1.00
81303	0.67	0.86	0.83	0.72
81593	0.96	0.82	0.84	0.95
81636	1	0.86	0.88	1.00
82065	0.92	0.85	0.86	0.91
83962	1	0.86	0.88	1.00
88696	0.99	0.77	0.81	0.99
89303	1	0.85	0.87	1.00
93025	0.98	0.84	0.86	0.98

Appendix 2B.

SUBJECT_ID	Sensitivity	Specificity	PPV	NPV
12122	0.94	0.93	0.93	0.94
21318	1.00	0.96	0.96	1.00
23657	0.97	0.96	0.96	0.97
40013	0.94	0.99	0.99	0.94
40057	1.00	0.97	0.97	1.00
43737	1.00	0.92	0.93	1.00
43961	1.00	0.99	0.99	1.00
44059	0.95	0.96	0.96	0.95
44789	1.00	0.97	0.97	1.00
44829	0.98	0.96	0.96	0.98
46320	1.00	0.95	0.95	1.00
48056	1.00	1.00	1.00	1.00
48388	1.00	0.99	0.99	1.00
49555	1.00	0.98	0.98	1.00
51078	1.00	1.00	1.00	1.00
51385	0.94	0.98	0.98	0.94
52641	1.00	0.81	0.84	1.00
54757	0.87	0.98	0.98	0.88
56069	1.00	0.98	0.98	1.00
56264	1.00	0.97	0.97	1.00
56307	1.00	0.92	0.93	1.00
58526	0.97	0.96	0.96	0.97
59085	1.00	0.87	0.88	1.00
60531	1.00	0.97	0.97	1.00
61619	1.00	1.00	1.00	1.00
62232	1.00	1.00	1.00	1.00
62795	0.88	0.98	0.98	0.89
63961	1.00	0.97	0.97	1.00
66571	0.99	0.96	0.96	0.99
67856	1.00	0.99	0.99	1.00
67996	1.00	1.00	1.00	1.00
68391	1.00	1.00	1.00	1.00

68453	1.00	0.96	0.96	1.00
72196	1.00	0.95	0.95	1.00
73299	0.62	0.92	0.89	0.71
75138	1.00	0.92	0.93	1.00
76930	1.00	1.00	1.00	1.00
77220	1.00	0.97	0.97	1.00
77927	0.60	0.92	0.88	0.70
80209	1.00	1.00	1.00	1.00
80350	1.00	0.85	0.87	1.00
80536	1.00	1.00	1.00	1.00
81303	0.64	0.91	0.88	0.72
81593	1.00	0.95	0.95	1.00
81636	1.00	0.99	0.99	1.00
82065	1.00	0.96	0.96	1.00
83962	1.00	0.97	0.97	1.00
88696	1.00	0.96	0.96	1.00
89303	1.00	0.99	0.99	1.00
93025	1.00	1.00	1.00	1.00
